# Seasonality and Cardio-Cerebrovascular Risk Factors for Benign Paroxysmal Positional Vertigo

**DOI:** 10.3389/fneur.2020.00259

**Published:** 2020-04-09

**Authors:** Zhentang Cao, Xingquan Zhao, Yi Ju, Meimei Chen, Yan Wang

**Affiliations:** ^1^Department of Neurology, Beijing Tiantan Hospital, Capital Medical University, Beijing, China; ^2^China National Clinical Research Center for Neurological Diseases, Beijing Tiantan Hospital, Capital Medical University, Beijing, China; ^3^Clinical Center for Vertigo and Balance Disturbance, Beijing Tiantan Hospital, Capital Medical University, Beijing, China

**Keywords:** benign paroxysmal positional vertigo, seasonality, cold season, temperature, cerebrovascular risk factors

## Abstract

**Background:** Benign paroxysmal positional vertigo (BPPV) is the most common cause of vertigo, especially in the elderly. Several studies have revealed a possible seasonality to BPPV. However, whether the seasonality of BPPV also exists in China is unclear. The characteristics of cardio-cerebrovascular risk factors for BPPV in the cold season have not yet been investigated.

**Objectives:** (1) To investigate the seasonality of BPPV; (2) To explore the relationship between cardio-cerebrovascular risk factors and seasonality of BPPV.

**Methods:** A retrospective observational study was performed in Beijing Tiantan Hospital from Jan 2016 to Dec 2018. The study included 1,409 new-onset BPPV patients aged 18–88 years. The demographic data, onset time, and medical history of BPPV were collected. The meteorological data, including temperature, atmospheric pressure, rainfall, and insolation, was obtained from Beijing Meteorological service. The *x*^2^ goodness of fit test was used to evaluate whether BPPV patients' numbers were significantly different among different months of the year. The Spearman correlation was used to detect the correlation between numbers of BPPV patients diagnosed monthly with each climatic parameter. The chi-square test for linear-by-linear association were used to investigate the relationship between cardio-cerebrovascular risk factor and seasonality of BPPV.

**Results:** November to next March is the top 5 months with higher BPPV patient numbers (*P* < 0.001). The numbers of BPPV diagnosed monthly were conversely correlated with temperature and rainfall (*r* = −0.736, *P* = 0.010; *r* = −0.650, *P* = 0.022, respectively), positively correlated with atmospheric pressure (*r* = 0.708, *P* = 0.010), but no significant correlated with insolation. BPPV in the cold season (including January, February, March, November, and December) had a higher proportion, accounting for 54.2% of all BPPV patients. Among BPPV patients with ≥2, 1, and none cardio-cerebrovascular risk factors, the cold season accounted for 57.0, 56.0, 49.8%, respectively. As the number of cardio-cerebrovascular risk factors increased, the proportion of patients in the cold season of BPPV increased (*P* = 0.025).

**Conclusions:** BPPV patients are seen more in the months with low temperature, low rainfall, and high atmospheric pressure. Compared with the non-cold season, BPPV patients have more risk factors for cardio-cerebrovascular diseases in the cold season.

## Introduction

Vertigo, a high frequency disease, imposes a rising burden on the health care system, aggravated by the aging of the population (1). Benign paroxysmal positional vertigo (BPPV) is the most common type of peripheral vestibular vertigo. It was caused by otoconia that migrate from the utricle to the semicircular canal or cupula. The clinical symptom is characterized by recurrent bouts of positionally triggered spinning vertigo. The lifetime prevalence of BPPV was estimated at 2.4%, the 1 year prevalence at 1.6% (2). At present, canalith repositioning maneuvers (CRM) are the primary treatment for BPPV, through the movement of the otoconia back into the utricle. Nevertheless, the recurrence rate of BPPV was high, a third to a half of patients have recurrences at 3 years, with most recurrences occurring in the first year (3). Due to the high recurrence, the quality of life, ability to perform activities of daily life have severely decreased. Meanwhile, it brings several complications, such as emotional disorder, sleep disorder, fall risk, and even death (4). Therefore, understanding the risk factors for BPPV is necessary for relieving symptoms and preventing recurrence.

Seasonality of patients with BPPV has been investigated. Whitman and Baloh (5) reported that the incidence of BPPV was significantly higher in the early spring months (March, April, May) in Boston. In Brazil, Pereira et al. found vertigo was more frequent in late winter- spring (6), but Zuma et al. showed more patients with BPPV are seen in consultation in the months with low solar radiation (March–September) (7). Korpon et al. demonstrated a association between barometric pressure and BPPV (8). Whether the seasonality of BPPV also exists in Chinese patients is unclear.

In addition to seasonality, the cardio-cerebrovascular risk factors (hypertension, diabetes, and hyperlipidemia) were associated with BPPV because of possible vascular damage to the inner ear (9). BPPV patients with hypertension and hyperlipidemia were at a higher risk of symptom recurrence (4). Patients with BPPV had a higher prevalence of coronary artery disease (10). Moreover, hyperglycemia and hyperinsulinemia are risk factors for the recurrence of BPPV (11). Cardiovascular and cerebrovascular diseases have a high incidence in the cold season. However, the relationship between cardio-cerebrovascular risk factors and seasonality of BPPV has not yet been investigated.

Therefore, we aimed to investigate the seasonality of BPPV in Chinese patients and to explore the relationship between cardio-cerebrovascular risk factors and seasonality of BPPV.

## Materials and Methods

### Meteorological Index

The study was conducted in Beijing Tiantan Hospital in China from January 2016 to December 2018. Beijing is located at latitude 39°56′N and longitude 116°20′E. It displays a typical temperate and monsoonal climate with four distinct seasons. The climate indexes included temperature, atmospheric pressure, rainfall, and insolation of the study period were retrieved from Beijing Meteorological service. Averages were calculated for each month across the 3 years study period. According to the temperature in Beijing, considering that from November to next March was recognized as the cold season. The meteorological indexes were documented to correlate these events with disease occurrence.

### Participants

The study was performed according to the Declaration of Helsinki guidelines, and written informed consent was obtained from all participants. The patients in our study only underwent standard treatment without additional interventions for research purposes, so no formal ethics approval was required. This study is a retrospective and exploratory study. The sample size of the previous study about seasonality and BPPV was ~207 (12) patients over the 3 years, 339 (13) patients over the 4 years, 956 (5) patients over the 5 years. About the cardiovascular risk factors and BPPV, the sample size of previous study was about 314 (14) patients over 4 years. At the same time, about 30–40 BPPV patients visited to our hospital every month. We retrospectively analyzed the data of 1,409 new-onset BPPV patients registered in the BPPV diagnosis and treatment registration database of Beijing Tiantan Hospital from Jan 2016 to Dec 2018. The diagnosis of BPPV met the criteria of BPPV established by the Barany Society (15). The all patients were first episode, received the definite diagnosis and canalith repositioning maneuvers (CRM). In order to describe the baseline clinical characteristics in detail, the patients were divided into five groups (aged 18–30 years, 31–44 years, 45–59 years, 60–80 years, ≥80 years) according to the age.

### Measurement Index

All patients underwent evaluations including demographic variables, potential risk factors, and neurological examination. The cardio-cerebrovascular risk factors included age ≥ 60 years, hypertension, hyperlipidemia, diabetes, coronary heart disease, migraine (16), and stroke. During the assessment, hypertension, hyperlipemia, diabetes, stroke, and coronary heart disease were defined according to International Classification of Diseases 10. Migraine was diagnosed on the basis of the International Headache Society (IHS) criteria (17). Sudden deafness was defined as a history of unilateral sensorineural hearing loss with sudden onset, without other prior otological histories (18). Probable Meniere's disease was defined according to the criteria defined by consensus among Barany Society, Japan Society for Equilibrium Research, EAONO, AAO-HNS, and Korean Balance Society (19).

### Statistical Analysis

Patients were divided into five different age groups (aged 18–30 years; 31–44 years; 45–59 years; 60–79 years; ≥80 years). All categorical variables are presented as frequency and percentage. Statistical significance between five groups was determined using a chi-squared test or Fisher's exact test. To evaluate whether patients' numbers were significantly different among different months of the year, the number of patients presented monthly was compared with the assumption of the equal number of patients diagnosed monthly. The comparison was analyzed by the *x*^2^ goodness of fit test. The Spearman's Rank Correlation Coefficient was used to study the correlation between overall numbers of patients diagnosed monthly with each climate parameter (temperature, atmospheric pressure, rainfall, and insolation) of each month of the year pooled from the years 2016–2018. The chi-square test for linear by linear association were used to determine the correlation between cardio-cerebrovascular risk factor and seasonality of BPPV. Statistical analysis was performed in SPSS 24.0 (IBM, Chicago, IL, USA). Graphs were delineated by using Prism 7.0 (GraphPad software, La Jolla, CA, USA). Values with *P* < 0.05 were regarded as statistically significant.

## Results

### Demographic Profiles of Participants

We collected 1,409 new-onset BPPV patients accepted definitive diagnosis in the Department of Neurology, Beijing Tiantan Hospital from Jan 2016 to Dec 2018. BPPV was found to be more frequent in female patients-947 (67.2%) against 462 (32.8%) males. The age varied from 18 to 88 years, with a median of 57.00 years (standard deviation = 12.91). The patients were divided into the five groups. Among groups, there was statistical difference in the common cardio-cerebrovascular risk factors, including hypertension, hyperlipemia, diabetes, coronary heart disease, and stroke. But no significant difference in the numbers with Meniere's disease, sudden deafness, and migraine was found (Table 1).

**Table 1:** Demographic information and clinical characteristics.

**Risk factors *n* (%)**	**18–30 y (*n* = 73)**	**31–44 y (*n* = 225)**	**45–59 y (*n* = 542)**	**60–79 y (*n* = 546)**	**≥80 y (*n* = 23)**	***P-*value**
Male	15 (20.5)	68 (30.2)	185 (34.1)	184 (33.7)	10 (43.5)	0.113
Hypertension	1 (1.4)	17 (7.6)	171 (31.5)	171 (31.3)	15 (65.2)	<0.0001
Hyperlipemia	0 (0)	9 (4.0)	99 (18.3)	110 (20.1)	6 (26.1)	<0.0001
Diabetes	0 (0)	1 (0.4)	27 (5.0)	59 (10.8)	6 (26.1)	<0.0001
Coronary heart disease	0 (0)	2 (0.9)	28 (5.2)	35 (6.4)	2 (8.7)	0.004
Stroke	0 (0)	2 (0.9)	9 (1.7)	24 (4.4)	1 (4.3)	0.008
Sudden deafness	5 (6.8)	9 (4.0)	16 (3.0)	27 (4.9)	1 (4.3)	0.256
Migraine	3 (4.1)	19 (8.4)	49 (9.0)	50 (9.2)	3 (13)	0.615
Meniere disease	0 (0)	0 (0)	2 (0.4)	4 (0.7)	0 (0)	0.622

### Seasonality of BPPV in Chinese Patients

**Figure 1:**
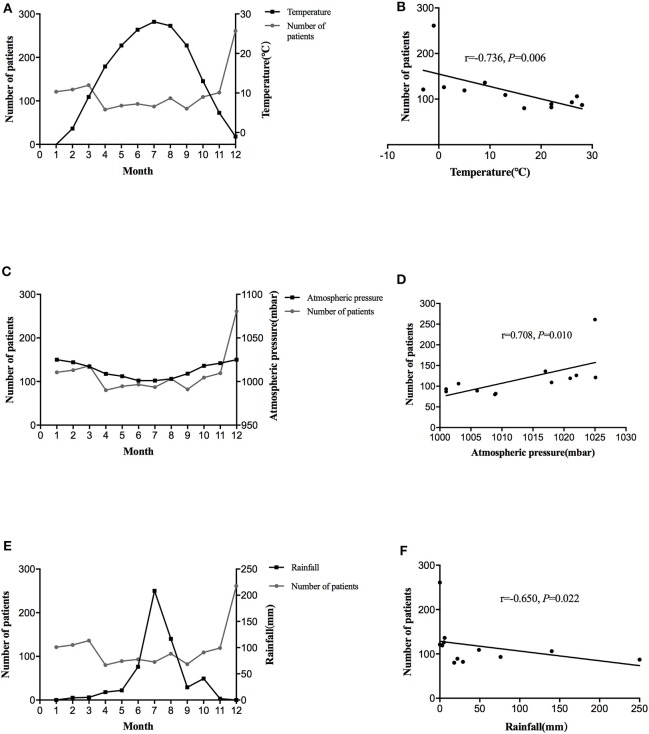
The distribution of average monthly diagnoses of BPPV and temperature over the 3 year period **(A)**. Association between the number of BPPV patients with temperature **(B)**. The distribution of average monthly diagnoses of BPPV and atmospheric pressure over the 3 year period **(C)**. Association between the number of BPPV patients with atmospheric pressure **(D)**. The distribution of average monthly diagnoses of BPPV and rainfall over the 3 year period **(E)**. Association between the number of BPPV patients with rainfall **(F)**.

As shown in Table 2, the distribution of patients is not equal in several months of the years. The overall monthly numbers of BPPV over the 3 years were significantly different (*P* < 0.001). The highest number of BPPV patients is in December, and the lowest number of BPPV is in April (Table 2, Figure 1).

**Table 2:** Overall monthly patients' distribution with percentage.

**Month**	**Overall number of patients in the years 2016, 2017, and 2018 *n* = 1,409**
Jan	121 (8.6%)
Feb	126 (8.9%)
Mar	136 (9.7%)
Apr	80 (5.7%)
May	89 (6.3%)
Jun	93 (6.6%)
Jul	87 (6.2%)
Aug	106 (7.5%)
Sep	82 (5.8%)
Oct	109 (7.7)
Nov	119 (8.4)
Dec	261 (18.5)
*P*-value	<0.001

The climatic indexes that were studied in relation to BPPV were the temperature, atmospheric pressure, rainfall, and insolation. Table 3 showed the mean values of these parameters. As shown in Table 3, the lower temperatures were in November-December and January-March (the cold season), and the higher were in April- October (Figure 1A). The atmospheric pressure was high in the cold season (November–December, January–March), less in the warm and hot months (Figure 1C). Similarly, the rainfall and insolation were low in the cold season (Figure 1E). The number of BPPV patients was conversely correlated with temperature and rainfall (*r* = −0.736, *P* = 0.006, Figure 1B; *r* = −0.650, *P* = 0.022; Figure 1F, respectively) and positively correlated with the atmospheric pressure (*r* = 0.708, *P* = 0.010; Figure 1D). Regarding the insolation, it was found to be conversely correlated to BPPV, yet this correlation was not significant (*r* = −0.203, *P* = 0.527).

**Table 3:** Average climatic indexes by months of the year in Beijing, China, 2016–2018.

**Month**	**Atmospheric pressure (mbar)**	**Average temperature (°C)**	**Rainfall (mm)**	**Insolation (h)**
Jan	1025.10	−3.03	0.10	197.13
Feb	1022.00	1.00	5.00	217.00
Mar	1017.00	9.00	6.00	238.00
Apr	1008.87	16.70	18.00	251.67
May	1006.00	22.00	22.00	269.00
Jun	1001.00	26.00	76.00	234.00
Jul	1001.00	28.00	250.00	164.00
Aug	1003.00	27.00	140.00	204.00
Sep	1009.00	22.00	29.00	210.00
Oct	1018.00	13.00	49.00	168.00
Nov	1021.00	5.00	3.00	187.00
Dec	1025.00	−1.00	0.07	188.00

### Relationship Between Cardio-Cerebrovascular Risk Factors and Seasonality of BPPV

BPPV in the cold season (including January, February, March, November, and December) had a high proportion, accounting for 54.2% of all BPPV patients. As shown in Table 4, among BPPV patients with ≥2, 1, and none cardio-cerebrovascular risk factors, the cold season accounted for 57.0, 56.0, 49.8%, respectively. As the number of cardio-cerebrovascular risk factors increased, the proportion of patients in the cold season of BPPV increased (*P* = 0.025).

**Table 4:** Relationship between cardio-cerebrovascular risk factors and seasonality of BPPV.

**The number of risk factors**	**Cold season (*n* = 763)**	**Non-cold season (*n* = 646)**	***x^**2**^***	***P-*value**
0	246 (49.8%)	248 (50.2%)		
1	255 (56.0%)	200 (44.0%)	5.000	0.025
≥2	262 (57.0%)	198 (43.0%)		

## Discussion

Our study showed that there is a seasonality to BPPV in China. The numbers of BPPV diagnosed monthly demonstrated a statistically significant converse correlation with temperature and rainfall, positive correlation with atmospheric pressure. As the number of cardio-cerebrovascular risk factors increased, the proportion of BPPV in the cold season increased.

In accordance with the present results, previous studies have revealed that the incidence of BPPV presents climatic variations in USA (5), UK (13), Iraq (12), and Brazil (7). Our results also showed in the cold season, namely in winter-spring (January, February, March, November, and December), had a high incidence in China. The numbers of BPPV diagnosed monthly had a statistically significant converse correlation with temperature. There were many possible explanations: (1) As we known, BPPV attacks when otoconia of the utricular macula become dislodged and freely floating otolithic debris moves into 1 or more of the semicircular canals. Calcium is the main component of otoconia crystals and Vitamin D is required for its regulation (20). A series of clinical observational studies showed that vitamin D levels were decreased in BPPV (13, 21, 22). Compared with the non-cold season, there is less sunlight time and a lower ultraviolet index, leading to decreased vitamin D levels in the cold season. Therefore, low vitamin D levels could cause the formation of calcium carbonate in endolymph. (2) At the same time, in the cold season, many people like the sedentary lifestyle rather than outdoor activities. The sedentary life may increase the incidence of bone demineralization and osteoporosis with possible increase in BPPV. (3) Other possible reasons for increased cases of BPPV shouldn't be neglected. Medical conditions that affect the inner ear, such as upper respiratory infections and allergies, have a higher occurrence of BPPV in winter and spring. Korpon et al. found an association between allergens and BPPV (8). Gacek et al. showed BPPV is associated with positive viral serology, particularly during certain months of the year, mainly in spring and autumn (23). These may also explain the relatively high incidence of BPPV in the cold season (winter-spring).

BPPV is the most common vestibular disease in females and the aged population. Women have doubled risk for BPPV than men (4, 24). Similarly, in the present study, there is a predominance of female sex, accounting for about 67.2% of BPPV patients. This female preponderance may be linked to hormonal factors (25). Estrogen deficiency has been shown to disturb the internal structure of the otoconia and their interconnection and attachment to the matrix (26). Oghalai et al. (27) carried out a study of unrecognized BPPV in elderly patients with the age of onset 45 to 60 years. The mean age is about 54.9 years, median age is about 57.00 years in this study. The semicircular canal function, as well as the otolith one, declines with age (28). Cardio-cerebrovascular risk factors, including hypertension, hyperlipidemia, diabetes, coronary artery disease, migraine, are considered as independent risk factors for the occurrence and recurrence of BPPV (9, 11, 29). The older patients have more cardio-cerebrovascular risk factors in our study. The possible reason was that the function of otolith got worse with organic changes caused by hypertension or diabetes which promote a diffuse vascular damage resulting in the atherosclerotic disease (29). An inner ear vascular damage caused by atherosclerosis can generate a progressive detachment of otoconia from the otolithic membrane. Especially in the cold season, accompanied by sympathetic nerve excitement and increased adrenaline secretion, people predisposed to faster heart rate, vasoconstriction, and higher blood pressure. Additionally, platelets, triglycerides, plasma fibrinogen, CRP, and other concentrations would also increase. These factors leading to an increase in the occurrence of ischemic vascular events caused the circulation disorder of inner ear. Our study also showed that the proportion of BPPV in the cold season is higher than that in the non-cold season. Furthermore, as the number of cardio-cerebrovascular risk factors increased, the proportion of BPPV in the cold season increased. Effective measures in the cold season including keeping warm and cardio-cerebrovascular risk factors control, may be helpful for prevention of BPPV.

In addition to the temperature, our study also showed that the number of BPPV patients was positively correlated with atmospheric pressure. Such findings confirm the association reported by Saeed and Omari (12) and Korpon et al. (8). The previous studies have demonstrated the pressure-sensitive nature of vestibular receptors and the presence of a valve-like structure that regulates endolymphatic pressure in the inner ear (30). The function of this valve was to regulate inner ear pressure with changes in atmospheric pressure (31). Transmission of this increased pressure by the inner ear space may lead to dislodgement of otoconia crystals leading to symptomatic BPPV. Also, there are studies that revealed that vestibular migraine (32) (VM) and Meniere's disease (33) (MD) have association with atmospheric pressure. Our study suggested a possible pathophysiologic link between the clinically observed coincidence of BPPV and VM or MD. The present study also showed that the number of BPPV patients was conversely correlated with rainfall and no significant correlation with insolation. The study about seasonality of vertigo (6) also demonstrated the above findings. The potential mechanism was not clear and warrant further evaluations.

Limitations of our study should be paid attention to: firstly, climatic variations may appear from year to year. This study endeavored to avert this by investigating 3 years of data. Secondly, it's a retrospective study based on clinical record and there was no follow-up information in this database. The specific and disease-related information was not available in this study. Therefore, further validated prospective studies are necessary to confirm our results. At last, some other cardio-cerebrovascular risk factors, such as behavior factors, were not available in this database. At present, this is the initial finding about the relationship between cardio-cerebrovascular risk factors and seasonality of BPPV in the cold season. Further prospective study is needed.

## Conclusions

BPPV patients are seen more in the months with low temperature, low rainfall and high atmospheric pressure. Compared with the non-cold season, BPPV patients have more risk factors for cardio-cerebrovascular diseases in the cold season.

## Data Availability Statement

The datasets generated for this study are available on request to the corresponding author.

## Ethics Statement

The study was performed according to the Declaration of Helsinki guidelines, and written informed consent was obtained from all participants. The patients in our study only underwent standard treatment without additional interventions for research purposes, so no formal ethics approval was required. Ethical review and approval was not required for the study on human participants in accordance with the local legislation and institutional requirements.

## Author Contributions

ZC conceived the study and design, conducted the experiment, and wrote the manuscript. XZ provided the data analysis and revised this manuscript. YJ conceived the study and design and edited the manuscript. YW and MC conducted acquisition of subjects and interpretation of data.

### Conflict of Interest

The authors declare that the research was conducted in the absence of any commercial or financial relationships that could be construed as a potential conflict of interest.
